# Temperature Dependence of the Magnetic Properties of IrMn/CoFeB/Ru/CoFeB Exchange Biased Synthetic Antiferromagnets

**DOI:** 10.3390/ma13020387

**Published:** 2020-01-14

**Authors:** Edoardo Albisetti, Giuseppe Scaramuzzi, Christian Rinaldi, Matteo Cantoni, Riccardo Bertacco, Daniela Petti

**Affiliations:** Dipartimento di Fisica, Politecnico di Milano, Via Giuseppe Colombo 81, 20133 Milano, Italy; giuseppe.scaramuzzi@mail.polimi.it (G.S.); christian.rinaldi@polimi.it (C.R.); matteo.cantoni@polimi.it (M.C.); riccardo.bertacco@polimi.it (R.B.)

**Keywords:** synthetic antiferromagnet, exchange bias, interlayer exchange coupling, vibrating sample magnetometry, CoFeB, thermally assisted magnetic scanning probe lithography, magnetron sputtering

## Abstract

Synthetic antiferromagnets (SAF) are widely used for a plethora of applications among which data storage, computing, and in the emerging field of magnonics. In this framework, controlling the magnetic properties of SAFs via localized thermal treatments represents a promising route for building novel magnonic materials. In this paper, we study via vibration sample magnetometry the temperature dependence of the magnetic properties of sputtered exchange bias SAFs grown via magnetron sputtering varying the ferromagnetic layers and spacer thickness. Interestingly, we observe a strong, reversible modulation of the exchange field, saturation field, and coupling strength upon heating up to 250 °C. These results suggest that exchange bias SAFs represent promising systems for developing novel artificial magnetic nanomaterials via localized thermal treatment.

## 1. Introduction

Synthetic antiferromagnets (SAFs) comprise ferromagnetic layers coupled antiferromagnetically through a non-magnetic spacer, by interlayer exchange coupling (IEC), which is essentially a Ruderman-Kittel-Kasuya-Yosida (RKKY) electronic coupling.

Starting from the first observation of the IEC in 1986 [1] and the subsequent study of the oscillatory behavior of IEC as a function of the spacer thickness [2,3], SAF systems played a fundamental role in the foundation of modern spintronics.

As in natural antiferromagnets, compensated SAFs present almost zero stray field and high stability. Furthermore, the interlayer coupling is typically orders of magnitude weaker than the exchange coupling between neighboring atoms in antiferromagnets, so that the manipulation of the magnetic order in SAF is easier. Some of the features that makes SAFs extremely interesting for applications is their wide applicability to in-plane as well out-of-plane magnetized materials, their large tunability via layer thickness and material composition, and the possibility to combine SAF and exchange bias by adding an antiferromagnetic layer to the stack. Throughout the years, SAFs were successfully used in spin-valves [4,5] and magnetic tunnel junctions [6,7,8,9] as reference layers, with applications as read heads in magnetoresistive [10,11] hard drives [12], magnetic random access memories (MRAM) [13,14,15], microwave oscillators [16] and magnetic biosensors [17,18,19,20,21,22]. More recently, SAFs have been proposed as base materials for racetrack memories [23], due to the higher domain wall velocity with respect to single ferromagnetic layers [24], and their capability to host a range of topological spin-textures [25]. SAFs have also raised considerable interest in the growing field of magnonics [26,27], due to the peculiar properties of spin-waves propagating in antiferromagnetically coupled bilayers [28,29]. Furthermore, recently it has been demonstrated that localized thermal treatments in combination with magnetic fields can be effectively used for manipulating the static and dynamic magnetic properties of exchange bias systems [30,31,32]. In this framework, studying the temperature dependence of their magnetic properties is of crucial importance. In this work, we perform a systematic study of the magnetic properties of exchange biased SAF systems grown via magnetron sputtering, varying the ferromagnetic layers thickness (from 25 nm to 45 nm) and the spacer layer thickness (from 0.6 nm to 0.7 nm). In particular, we use vibration sample magnetometry (VSM), for measuring quantitatively the hysteresis loops as a function of the in-plane field angle and temperature, ranging from room temperature up to 250 °C. Then, from the hysteresis loops we extract the exchange field, saturation field, saturation magnetization and exchange constant, and study their dependence on temperature. This work sheds light on the temperature behavior of exchange biased synthetic antiferromagnets, allowing the design and implementation of novel methodologies for the thermally assisted control of their magnetic properties. 

## 2. Materials and Methods

The samples were grown on Si/SiO_2_ (100 nm) substrates by employing an AJA Orion8 magnetron sputtering system (AJA International, Scituate, MA, USA) with a base pressure below 1 × 10^−8^ Torr. All the materials were sputtered in the DC mode from single stoichiometric targets, using the parameters listed in Table 1. Note that two different conditions were employed for Ru: 3 mTorr for Ru films exploited as a capping layer and 5 mTorr for Ru films used as interlayer in the synthetic antiferromagnet. Indeed, the lower deposition rate at a pressure of 5 mTorr allows a better control on the Ru thickness, fundamental to obtain a sizable interlayer exchange coupling of the two ferromagnets. 

Before the deposition, the substrates underwent a soft etch in the same chamber at an Argon pressure of 3 mTorr and an RF power of 20 W for 5’, in order to clean the surface. During the growth, a 30 mT magnetic field (*H_G_*) was applied in the sample plane for setting the magnetocrystalline uniaxial anisotropy direction in the Co_40_Fe_40_B_20_ layer and the exchange bias direction in the as-grown sample.

After the growth, the samples underwent an annealing at 250 °C for 5 min, which promoted the crystallization of the amorphous CoFeB and the IrMn layers.

**Figure 1:**
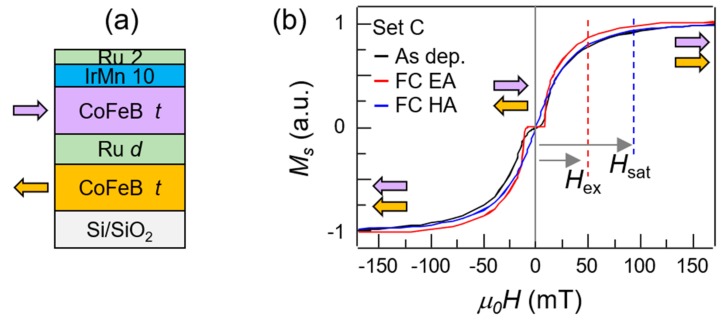
(**a**) Sketch of the CoFeB/Ru/CoFeB/IrMn/Ru synthetic antiferromagnet stacks grown via magnetron sputtering. *t* indicates the thickness of the CoFeB layers, ranging from 25 nm to 45 nm. *d* indicates the thickness of the Ru coupling layer, ranging from 0.6 to 0.7 nm; (**b**) room-temperature vibrating sample magnetometer hysteresis loop of sample C (CoFeB 35 nm, Ru 0.6 nm) in the as deposited sample measured along the easy axis (black line) and the sample after a field cooling at 250 °C measured along the easy axis (red line) and hard axis (blue line). The colored arrows indicate the direction of the magnetization of top (purple) and bottom (orange) layers. *H_ex_* and *H_sat_* indicate the exchange field and saturation field related to the annealed sample, respectively.

Different sets of samples were studied, consisting of the stacks shown in Figure 1a, namely Si/SiO_2_/Co_40_Fe_40_B_20_ *t*/Ru *d*/Co_40_Fe_40_B_20_ *t*/IrMn 10/Ru 2 (dimensions in nm) varying the thickness of the CoFeB ferromagnetic layers (*t*) and that of the non-magnetic interlayer Ru (*d*). The specific CoFeB and IrMn stoichiometry was chosen because of their widespread use in magnetic tunnel junctions, the low spin-wave damping in Co_40_Fe_40_B_20_ and the high exchange bias strength. The exact composition of the studied samples is reported in Table 2, using the same coding which will be used in the subsequent discussion.

The hysteresis loop of the films was measured via a vibrating sample magnetometer (VSM; EZ-9, Microsense, Lowell, MA, USA) as a function of the sample temperature, from RT to 250 °C, and of the direction of the magnetic field in the film plane. The VSM was used also for performing the initial annealing and field cooling of the samples.

## 3. Results and Discussion

### 3.1. Room-Temperature Magnetic Characterization and Angular Hysteresis Loops

**Figure 2:**
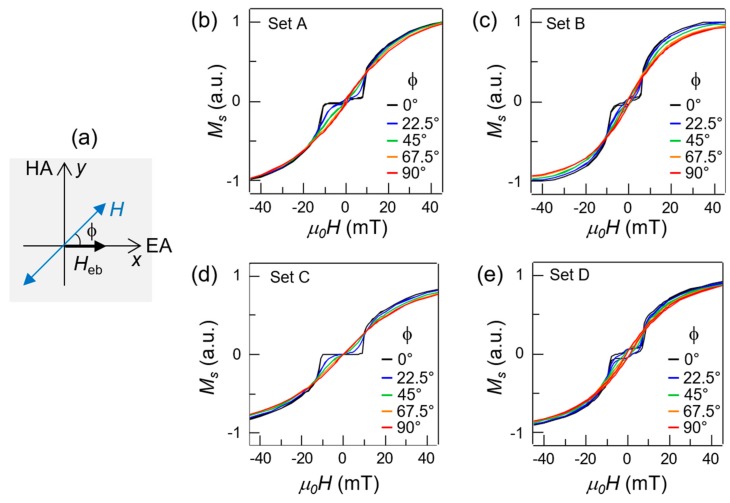
Room temperature hysteresis loops as a function of the field angle. (**a**) Sketch of the experimental configuration for the measurements. The thick arrows indicate the direction of the exchange bias field, set during field cooling (*H_eb_*) along the *x* axis direction. *ϕ* indicates the angle of the external applied field (*H*) with respect to the exchange bias direction, in the plane of the film. (**b**–**e**) Hysteresis loops measured via vibration sample magnetometry (VSM) at room temperature as a function of the angle *ϕ* (color-coded) for set A, B, C, and D respectively.

Figure 1b shows the VSM hysteresis loops along the easy axis acquired at room temperature of set C (35 nm CoFeB, 0.6 nm Ru) for the as deposited sample, black line, and after the field cooling, red line. The field cooling was performed by heating the sample up to 250 °C and subsequently cooling it down to room temperature while applying a static 400 mT external magnetic field in the sample plane, in the same direction of the field applied during the growth, *H_G_*. The field cooling sets the direction of the exchange bias, and therefore the pinning direction of the magnetization of the top CoFeB layer. The direction of the magnetization of the top (bottom) CoFeB layers as a function of the external field is indicated by the purple (orange) arrows. The hysteresis loop displays the characteristic plateau at low field, which is a signature of robust antiferromagnetic coupling between the two CoFeB layers, induced by the interlayer exchange coupling across the thin Ru layer. The plateau is also a signature of the presence of the anisotropy of the film, which is further confirmed by the angle-dependent measurement of Figure 2. The two main contributions are the magnetocrystalline anisotropy and the exchange bias set during the growth. The two lobes of the loop are related to the rotation of the top (negative fields) and bottom (positive fields) CoFeB layer. The saturation field *H_sat_* and the exchange field *H_ex_* are indicated in figure for the measurement after field cooling. In particular, *H_ex_* was extracted from the measurement as the halfway point between the end of the low-field plateau and the saturation field. In high external fields, the antiferromagnetic coupling is overcome by the Zeeman field, which saturates the magnetization in the direction of the applied field. Upon decreasing the magnetic field, the magnetizations rotate coherently and gradually reach the antiparallel alignment. The well-defined plateau in the annealed samples, with respect to the as-deposited samples, mainly arises from setting the unidirectional anisotropy of exchange bias via field cooling.

Figure 2 shows the room temperature hysteresis loops after field cooling as a function of the angle of the external field applied in the plane of the sample. Panel (a) shows the geometry of the measurement. In particular, *ϕ* is the angle between the direction of the exchange bias *H_eb_*, set during the field cooling, and the direction of the external field applied during the measurement *H*. Specifically, the easy magnetization axis (EA) is given by *ϕ* = 0° and the hard axis (HA) is for *ϕ* = 90°. The measurements were performed in the same conditions on all the sets with a step of 22.5° from *ϕ* = 0° to *ϕ* = 90°, color-coded. In all the sets, the loops feature the characteristic two lobes and low-field plateau along the EA, and the elongated loop along the HA. For intermediate angles, the loops assume a mixed EA and HA character, with the low field plateau progressively tilting to higher derivatives. Some samples show at low field a small hysteresis loop, probably due to slightly non-compensated ferromagnetic structures. The origin of non-compensation can be ascribed to the small variation of the CoFeB deposition conditions, or possibly to the influence of the surface roughness of one of the two ferromagnetic layers [33,34].

### 3.2. Temperature Dependence of the Magnetic Properties

**Figure 3:**
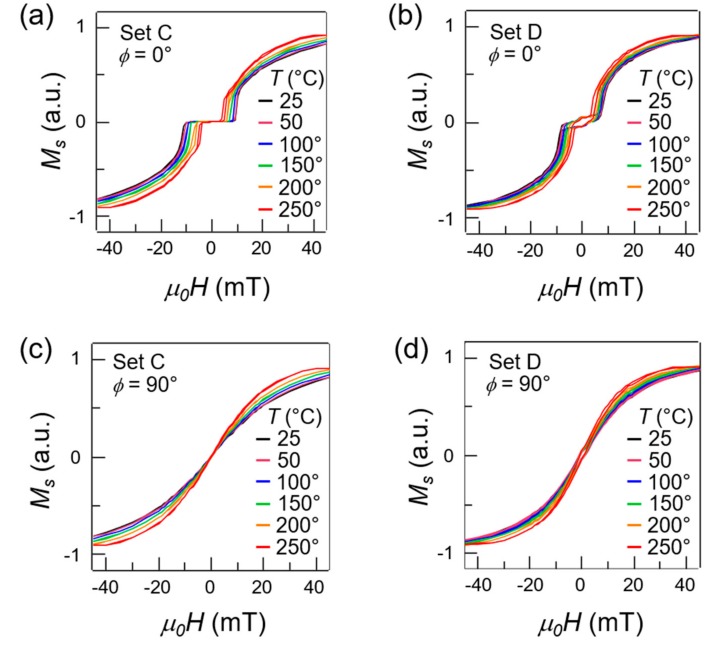
(**a**,**b**) Hysteresis loops of set C and set D along the easy-axis (*ϕ* = 0°) measured as a function of temperature (color-coded) ranging from 25 °C to 250 °C; (**c**,**d**) Hysteresis loops of set C and set D along the hard-axis (*ϕ* = 90°) measured as a function of temperature (color-coded) ranging from 25 °C to 250 °C.

Figure 3 shows the hysteresis loops of sets C and D along the easy axis (*ϕ* = 0°, panels (a–b)) and hard axis (*ϕ* = 90°, panels (c–d)), as a function of temperature, ranging from room temperature up to 250 °C, color-coded. Regarding the EA loops, three main effects of temperature are observed. First, a reduction of the plateau field with increasing temperature, from ~9 mT at room temperature down to ~4 mT at 250 °C in set C, and from ~7 mT at room temperature down to ~4 mT at 250 °C in set D. The decrease of the plateau was mainly due to the weakening of the unidirectional anisotropy set by the exchange bias while approaching the blocking temperature, combined with a reduction of the interlayer coupling strength, as the dependence of the magnetocrystalline anisotropy on the temperature in this ranges is much weaker [35]. Second, a reduction of the saturation field *H_sat_* and exchange field *H_ex_*, which was consistent with a reduction in the interlayer coupling with temperature. Third, as expected [36,37] we observe a slight reduction of the saturation magnetization *M_s_* with increasing temperature. All these effects were however reversible, provided that the same exchange bias direction and strength was retrieved at the end of the heating process, e.g., (as performed in our experiments) by applying an external saturating field in the direction of the exchange bias during the cooling process.

**Figure 4:**
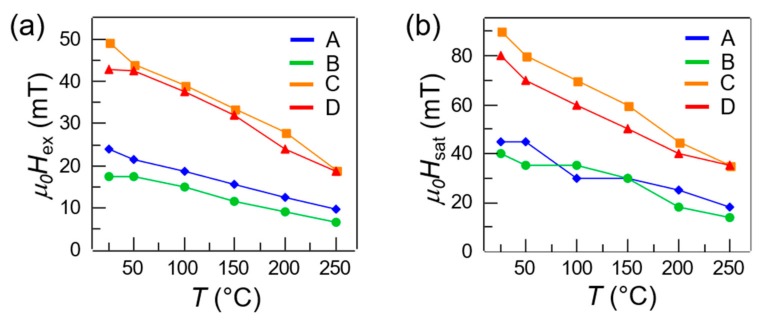
(**a**,**b**) Exchange field *μ_0_H_ex_* and saturation field *μ*_0_*H_sat_* as a function of temperature for sets A–D (color-coded).

For analyzing quantitatively the characteristics of the hysteresis loops, in Figure 4 we plotted the exchange field *H_ex_* (a) and saturation field *H_sat_* (b) as a function of the temperature for all the sample sets (A–D). From the room temperature data (at *T* = 25 °C) of both panel (a) and panel (b), we observed higher *H_ex_* around 45 mT and *H_sat_* around 85 mT in sets C–D, and lower *H_ex_* around 20 mT and *H_sat_* around 40 mT in sets A–B. This was consistent with the oscillatory nature of the interlayer coupling as a function of the Ru thickness, which was 0.6 nm in sets C–D and 0.7 nm in sets A–B. It is worth noting that, the Ru thickness being equal, slightly higher exchange and saturation fields were observed in the sets characterized by thinner CoFeB layers (set A, 25 nm and set C, 35 nm). This is coherent with the interfacial nature of the antiferromagnetic interlayer coupling, which is inversely proportional to the film thickness.

Regarding the temperature dependence of the exchange and saturation fields, we observed an overall reduction of both parameters with increasing temperature, in all sample sets. In particular, both *H_ex_* and *H_sat_* displayed a reduction of about 60% from room temperature to 250 °C. This behavior is particularly interesting in the framework of thermally assisted magnetic writing schemes, such as TAMR (thermally assisted magnetic recording) or tam-SPL (thermally assisted magnetic scanning probe lithography), where local heating in combination with external fields is used for writing magnetization patterns. Note that, at the same time, the reduction of the plateau field was around 50%. 

**Figure 5:**
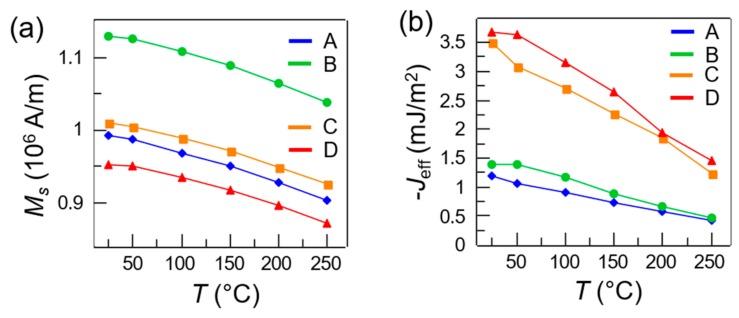
(**a**) Saturation magnetization *M_s_* as a function of temperature for set A–D (color-coded); (**b**) Coupling strength *J_eff_* as a function of temperature for set A–D (color-coded).

In Figure 5, we studied the saturation magnetization (a) and the interlayer coupling strength (b) as a function of temperature. *M_s_* measured at room temperature is 0.9–1.1 × 10^6^ A/m in all sets, which is in agreement with values found in the literature [38,39] for CoFeB films with the same stoichiometry. The *M_s_*, which lowers with increasing temperature due to magnon excitation [40], was calculated normalizing the quantitative VSM measurements by the magnetic volume of each sample. The slight variations observed across different samples are due to non-idealities in the sample shape. In order to study the dependence of the coupling coefficient on the temperature, we calculated the total effective coupling strength *J_eff_* = *H_ex_M_s_t*, where *H_ex_* is the exchange field (see Figure 1b), *M_s_* is the saturation magnetization and *t* is the sample thickness. The physical origin of such dependence can be ascribed to both interfacial effects, spacer-related mechanisms or effects arising in the magnetic layers [41]. In panel (b) we plot *J_eff_* as a function of temperature for all the sets. As previously noted, sets A and B (Ru 0.7 nm thick) feature a lower *J_eff_*~ 1.25 mJ/m^2^ with respect to sets C and D (Ru 0.6 nm thick), which feature *J_eff_*~ 3.5 mJ/m^2^ at room temperature, due to the different Ru interlayer thickness, consistently with the expected oscillatory behavior [2]. Noteworthy, *J_eff_* was reduced reversibly by 60–65% from room temperature to 250 °C with only slight variations across different sets, suggesting that even mild heating treatment could significantly and temporarily alter the SAF coupling strength, with high potential for applications. These results were consistent with those found in similar system, namely synthetic antiferromagnets based on thin ferromagnetic layers with in plane and perpendicular to the plane magnetic anisotropies [35,42]. The theoretical explanation of the decrease of interlayer exchange coupling with the temperature can be found in [41].

## 4. Conclusions

In this work, we studied the temperature and angular dependence of the magnetic properties of exchange biased synthetic antiferromagnets, in the case of compensated in-plane magnetized layers with thickness ranging from 25 nm to 45 nm. Noteworthy, we observed a strong reversible reduction of the exchange field, saturation field and antiferromagnetic coupling strength with a mild heating treatment (up to 250 °C). These results are particularly interesting in the framework of heat-assisted magnetic writing methods such as thermally assisted magnetic scanning probe lithography (tam-SPL), where localized heating in combination with external fields were used for reversibly writing the magnetic properties of thin-film materials. These results suggest that SAFs represent promising systems for developing novel artificial magnetic nanomaterials via localized thermal treatment.

## Figures and Tables

**Table 1:** Magnetron sputtering parameters.

Material	Power (W)	Ar Pressure (mTorr)
Ru	50	3
Ru SAF	50	5
Co_40_Fe_40_B_20_	58	4
Ir_22_Mn_78_	50	3

**Table 2:** List of the studied sample sets and corresponding stack compositions.

Set Name	Stack Compositions (Dimensions in nm)
A	Si/SiO_2_/CoFeB 25/Ru 0.7/CoFeB 25/IrMn 10/Ru 2
B	Si/SiO_2_/CoFeB 35/Ru 0.7/CoFeB 35/IrMn 10/Ru 2
C	Si/SiO_2_/CoFeB 35/Ru 0.6/CoFeB 35/IrMn 10/Ru 2
D	Si/SiO_2_/CoFeB 45/Ru 0.6/CoFeB 45/IrMn 10/Ru 2
